# Racial, ethnic, and income disparities in air pollution: A study of excess emissions in Texas

**DOI:** 10.1371/journal.pone.0220696

**Published:** 2019-08-02

**Authors:** Zhengyan Li, David M. Konisky, Nikolaos Zirogiannis

**Affiliations:** School of Public and Environmental Affairs, Indiana University, Bloomington, Indiana, United States of America; University of Southern California, UNITED STATES

## Abstract

**Objective:**

Excess emissions are pollutant releases that occur during periods of startups, shutdowns or malfunctions and are considered violations of the U.S. Clean Air Act. They are an important, but understudied and under-regulated, category of pollution releases given their frequency and magnitude. In this paper, we examine the demographic correlates of excess emissions, using data from industrial sources in Texas.

**Methods:**

We conduct two complementary sets of analyses: one at the census tract level and one at the facility level. At the census tract level, we use a multinomial logit model to examine the relationships between racial, ethnic, and income characteristics and the incidence of excess emissions. At the facility level, we first estimate a logit model to examine whether these characteristics are associated with facilities that emit excess emissions, and then, conditional on the presence of excess emissions, we use ordinary least square regression to estimate their correlation with the magnitude of releases.

**Results:**

Across our analyses, we find that the percentage of Black population and median household income are positively associated with excess emissions; percentage of college graduate, population density, median housing value, and percentage of owner-occupied housing unit are negatively associated with excess emissions. We, however, have not found a clear and significant relationship between the percentage of Hispanic population and excess emissions.

## Introduction

Air quality remains an important challenge for millions of people in the United States. According to the most recent data from the U.S. Environmental Protection Agency (EPA), more than 130 million people currently live in an area of the country that does not meet national ambient air quality standards established under the U.S. Clean Air Act (CAA) [1]. One important cause of persistent air quality problems are industrial facilities, which remain large sources of criteria air pollutants such as particulate matter and sulfur dioxide and toxic substances such as mercury and benzene.

The CAA is designed and implemented to directly regulate the release of these types of air pollutants when they are emitted through the regular operations of power plants, oil refineries, chemical manufacturers, and other facilities. These same facilities, however, also often emit excess emissions (sometimes referred to as upset emissions), which are defined by the EPA as emissions “that occur during the startup, shutdown, malfunction or other modes of sources operation, i.e., emissions that would be considered violations of the applicable emissions limitations but for an impermissible automatic or discretionary exemption from such emissions limitation” [2]. Excess emissions often result from unexpected or unavoidable circumstances, such as when a facility must shut down operations due to a power outage or natural disaster, or if a pollution control device malfunctions due to poor maintenance.

Excess emissions are important for several reasons. First, recent work suggests that releases are frequent and often times large in magnitude, in some instances surpassing on an annual basis the total emissions from a facility’s normal (or routine) operations [3, 4]. Second, excess emissions events can result in the release of both criteria and toxic pollutants, thereby affecting both environmental quality and creating risks for a multitude of adverse health effects. Third, excess emissions are generally under-regulated, even though the EPA characterizes them as CAA violations when they go beyond a facility’s permitted limits. Although states implementing the CAA are obligated to regulate this category of emissions, most have routinely granted automatic exemptions (provided a list of criteria are met) rather than pursue enforcement measures. In addition, many states have historically allowed facilities to make affirmative defense claims so long as they can document that an excess emissions event was a result of truly unavoidable circumstances and did not contribute to (or cause) a violation of the National Ambient Air Quality Standards (NAAQS). If the state regulator grants the affirmative defense request, then the facility cannot be held liable for civil penalties at the state level.

EPA policy regarding excess emissions is in a state of flux. Prompted by a lawsuit, the EPA issued a notice in 2015, asking 36 states to revise the language in their State Implementation Plans (SIPs), dictating how excess emissions are regulated, in order to bring those SIPs in compliance with the CAA [2]. The 2015 SIP specifically directed states to remove automatic exceptions and affirmative defense provisions from their SIPs. Soon after the EPA’s SIP Call was published in the Federal Register, several states sued the EPA, and, as a result, the 2015 SIP Call was held in abeyance [5]. In recent months, the EPA has engaged in a deregulatory effort through its regional offices (Region 6 and Region 4) with the goal of creating a laxer enforcement framework around excess emissions [5,6]. In light of this deregulatory effort, it is especially important to better understand the geographic distribution of excess emissions, and which segments of the U.S. population are most affected.

Despite their frequency and magnitude, and a historically lax regulatory approach, excess emissions are under-studied. One reason for a lack of scholarly attention may be limited data. Information on excess emissions is maintained at the state level only, as federally maintained datasets like the National Emissions Inventory and the Toxics Release Inventory (TRI) do not include them. The TRI includes data on fugitive emissions defines as: “equipment leaks, releases from building ventilation systems and evaporative losses from surface impoundments and spills” [7]. However, this category of emissions is distinct from excess emissions. Even at the state level, data on excess emissions are sparse. Only three states–Texas, Oklahoma and Louisiana–keep systematic records in a way that make the data meaningfully accessible.

The limited literature to date has studied two dimensions of excess emissions. First, atmospheric scientists have investigated the degree to which excess emissions impair air quality [8–12]. A second stream of research has explored patterns of excess emissions across states and industries [4, 13, 14]. In the most comprehensive analysis to date, Zirogiannis, Hollingsworth, and Konisky [3] analyze patterns of excess emissions across sectors, facilities, multiple pollutants, and over more than a decade of time from 2004–2015.

Collectively, this literature has demonstrated that excess emissions occur regularly, are sometimes of very large magnitude, and can severely diminish air quality. Given decades of research that links poor air quality and adverse health outcomes [15–20], the consequences of excess emissions may be significant. In Texas alone, and solely considering excess emissions of sulfur dioxide and nitrogen oxides (that result in secondary particulate matter), Zirogiannis, Hollingsworth, and Konisky [3] estimated the health damages to be approximately $150 million annually. When accounting for health damages by excess emissions of additional pollutants (such as ammonia, VOCs and directly emitted PM_10_) the value of monetized damages from premature mortality and morbidity increases to $250 million annually [21]. Hollingsworth, Konisky and Zirogiannis [21] use mortality data at the county level in Texas and find that excess emissions are responsible for 42 deaths per year in people 65 years and older.

The extant literature on excess emissions has not yet studied who might be most at risk to their associated impacts to air quality. This is an important lacuna. To the extent that the types of industrial facilities most prone to releasing excess emissions are not uniformly distributed across geographic space, the effects may disproportionately fall on some communities. In particular, the well-established environmental justice literature has regularly found evidence that, for example, African-Americans, Hispanics, Native Americans, and individuals living in poverty are more likely to host waste disposal facilities and major sources of pollution and to face exposure to higher emissions and poorer environmental quality [22–30]. The evidence is not uniform, and there remain important, unresolved debates about the causal mechanism(s) underlying these patterns [31–33]. Nevertheless, the presence of these disparities has generated social mobilization among affected communities [34, 35], and at least some limited response from federal, state, and local government agencies [33, 36–39].

This paper is the first to analyze the demographic and socioeconomic correlates of excess emissions. Dissimilar to many studies in the environmental justice literature, our objective is to identify correlations between both the presence and magnitude of excess emissions, rather than the factors that correspond to the siting of facilities in communities of color and low income.

Questions around facility siting are certainly important, and past studies have shown that race, ethnicity, and income are often correlated with the location of pollution sources [22, 23]. The causal mechanism(s) behind these associations are still in dispute, but uncertainty about the social, economic, and political processes that lead to correlations between pollution and demographic attributes does not mean that their existence is unimportant [40]. Disparities, regardless of their cause are essential to document from an environmental justice perspective, and particularly so given that the category of pollution at issue are under-regulated.

Specifically, here we seek to answer the question of whether excess emissions from industrial sources are associated with the racial, ethnic, and income attributes of communities. Studying data on excess emissions from large industrial sources in Texas, we find that the percentage of Black population and median household income are positively associated with excess emissions. The correlation between the percentage of Hispanic population and excess emissions is more ambiguous, but we do find that excess emissions are more likely to occur in facilities that are located in communities with higher percentages of Hispanic population. These findings regarding race and ethnicity are consistent with existing literature on environmental justice and pollution disparities, but the results regarding income are less clear, which may suggest idiosyncrasies in industrial siting patterns in Texas.

## Materials and methods

Our empirical analysis examines the correlations between excess emissions and demographics. This section describes the data and statistical models we use in the analysis.

### Data

The excess emissions data we use were obtained from the Texas Commission on Environmental Quality (TCEQ)’s Emissions Inventory (EI) dataset. The EI dataset includes annual totals for more than 2000 pollutants released from major sources (i.e., CAA Title V facilities) in Texas. Facilities report the following annual amounts in the EI data set: (1) routine emissions (i.e., permitted emissions); (2) emissions events (EE); and (3) emissions attributed to scheduled startup, shutdown or maintenance (SMSS) events. Taken together, EE and SMSS emissions constitute the total amount of excess emissions. The TCEQ [41] defines an Emissions Event as “any upset event or unscheduled maintenance, startup or shutdown activity …that results in unauthorized emissions.” Emissions events result in releases from a stack as opposed to fugitive emissions that “could not reasonable pass through the stack.” An SMSS event is a scheduled event that is expected to exceed authorized emissions levels and for which a facility is required to provide prior notification and submit a final report to the TCEQ [41].

The EI includes information from EE and SMSS events regardless of whether or not the authorized emissions threshold was exceeded. From that perspective, the excess emissions definition of the EPA (discussed above) does not align exactly with the way EE and SMSS emissions data are reported in Texas’ EI. However, in the absence of the ability to distinguish between reportable and non-reportable emissions in the EI, we use the totality of EE and SMSS emissions information, and simply note that our data likely overestimate unauthorized emissions. That said, there is also a source of underestimation in the data since only Title V facilities are required to report to the EI, meaning that excess emissions released from smaller facilities are excluded completely. While smaller (non-Title V) facilities are required to report their excess emissions events in TCEQ’s Air Emissions and Maintenance Events (AEME) database, which is distinct from the EI, the AEME dataset is compiled differently from the EI, and it is not possible to use information from both datasets in our analysis. The dataset used in this study is a compilation of these events from 2000 to 2010. Emissions across pollutants are aggregated to the facility-year level and census tract-year level respectively by categories of excess emissions (EE+SMSS) and routine (permitted) emissions.

The demographic data we use were obtained from the 2000 and 2010 U.S. Decennial Censuses, supplemented with the 2012 American Community Survey (ACS). We extract seven key demographic characteristics at the census tract level: percentage of Black population, percentage of Hispanic population, population density, percentage of college graduates among population 25 year and over, median household income, median housing value, and percentage of owner-occupied housing unit. Because the 2010 Census does not include census tract level information on college graduates, median household income, median housing value, and percentage of owner-occupied housing unit, we use the 5-year estimates from the 2012 ACS. Specifically, since the ACS does not have a sufficiently large sample size to provide accurate yearly estimates at the census tract level, it uses samples from 2008 to 2012 to construct the 5-year census tract estimates for 2012. Following the suggestion of Census Bureau, we use the 2012 5-year estimates to substitute the missing information for 2010, the middle year of the period from 2008 to 2012. For the interim years between 2000 and 2010, we use linear interpolation for the census tract level demographic variables.

### Statistical methods

The distribution of excess emissions in the data presents two analytical challenges. First, there are a large number of zeros in the excess emissions data. Specifically, in the study period, more than 60% of the facilities in Texas did not report having any excess emissions. Second, there are outliers in the excess emissions data as a few extreme events contribute the majority of the excess emissions in a certain year. For instance, Zirogiannis, Hollingsworth, and Konisky [3] find that, for most years, excess emissions from the top 5% of events constitute more than 50% of the total excess emissions.

The distribution of excess emissions renders a simple OLS regression of all the data at the facility level inappropriate for our analysis. We could alleviate the problem of outliers by using the logarithm transformation of excess emissions. This, however, requires excluding observations with zero excess emissions from our analysis, which could lead to potential sample selection bias. Given the data structure, we pursue two complementary empirical strategies: 1) a multinomial logit specification conducted at the census tract level; and 2) a combination of a logit and OLS regression at the facility level. We discuss each, as well as their limitations, in turn.

#### Census tract level analysis

For the multinomial analysis at the census tract level, we categorize all census tracts in Texas into three types: census tracts with neither routine emissions nor excess emissions (Type 1), census tracts with routine emissions but no excess emissions (Type 2), and census tracts with both routine emissions and excess emissions (Type 3). The multinomial model examines the correlations between demographics and the probabilities of being in one of these types of census tracts.

More specifically, we estimate the model:

$$Multinomial\;Logit:\;y_{it}=\alpha +\beta X_{it}+\gamma_{t}+\varepsilon_{it},$$

where *y*_*it*_ is census tract type, consisting of the three categories discussed above, for census tract *i* in year *t*, *X*_*it*_ is a vector of census tract demographic characteristics (percentage of Black population, percentage of Hispanic population, population density, percentage of college graduate, median household income, median housing value, and percentage of owner-occupied housing unit), γ are year dummies, ε is an error term.

Table 1 shows the frequencies of each type of census tract across all years. The dataset is dominated by Type 1 tracts, with 84% of the observations having neither routine emissions nor excess emissions (i.e. no Title V facility is located in those tract-years). About 9% of the tract-year observations have some amount of routine emissions but no excess emissions and another 7% of the observations are tract-years with both routine emissions and excess emissions. (See **Table A** in **S1 File** for the descriptive statistics of the independent variables used in the census tract analysis.)

**Table 1:** Types of census tracts.

Census Tract Type	Frequency	Percent
Type 1 (Routine Emissions = 0, Excess Emissions = 0)	47,746	84.45%
Type 2 (Routine Emissions > 0, Excess Emissions = 0)	4,880	8.63%
Type 3 (Routine Emissions > 0, Excess Emissions > 0)	3,910	6.92%
Total	56,536	100.00%

Note: Values in Frequency column represent the number of census tract-year observations.

#### Facility level analysis

Our facility level analysis consists of two components. First, we estimate a logistic regression to model the binary outcome of whether or not a facility has excess emissions. This specification allows us to characterize the relationship between the presence of excess emissions and the demographic features of the areas in close proximity to a facility. Second, we estimate an OLS regression to model the level of excess emissions, conditioning on a facility having any positive amount of excess emissions. This approach enables us to examine the associations between demographic characteristics of the nearby area with the magnitude of excess emissions. It does, however, make an important assumption by claiming that a zero excess emissions threshold can be used to explain differences in magnitudes across different neighborhoods surrounding each facility. One could envision alternative approaches where that threshold has any positive value. Due to the lack of any theoretical or empirical prior as to what that threshold is, we believe that assuming a zero threshold is a valid assumption.

More precisely, we estimate the following two models:

$$Logit:\;y_{1it}=\alpha_{1}+\beta_{1}X_{it}+s_{1i}+\gamma_{1t}+\varepsilon_{1it}$$

$$OLS:\;y_{2it}=\alpha_{2}+\beta_{2}X_{it}+s_{2i}+\gamma_{2t}+\varepsilon_{2it},$$

where *y*_*1it*_ is a binary variable of whether or not facility *i* had excess emissions in year *t*, *y*_*2it*_ is the level of excess emissions (log transformed) for facility *i* in year *t* conditional on the facility having some amount of excess emissions in year *t*. All right-hand side variables are the same across the two models: *X*_*it*_ represents a vector of facility demographic characteristics (percentage of Black population, percentage of Hispanic population, population density, percentage of college graduate, median household income, median housing value, and percentage of owner-occupied housing unit) and facility routine emissions, *s*_*1i*_ and *s*_*2i*_ are industry dummies (based on the Standard Industry Classification codes) for the two models respectively, γ_1t_ and γ_2t_ are year dummies for the two models respectively, and ε is an error term.

To construct the facility-level demographic information, we use an areal apportionment method, which is common in the environmental justice literature [42]. Specifically, we first use Geographic Information Systems to create a circular buffer with a 1-mile radius (and a 3-mile radius for robustness check) from each facility (the EI data contains latitude and longitude, enabling us to precisely locate facilities geographically). Second, we overlay the buffer layer over the census tract layer to create intersections between the two layers. Third, assuming population and households are evenly distributed within a census tract, we calculate for each intersection the total population and the population of different races, ethnicities, and education levels, and the number of households, based on the proportion of the area of each intersection in the area of the census tract that the intersection belongs to. Fourth, we calculate for each circular buffer the total population and the population of different races, ethnicities, and education levels, and the number of households by respectively summing up the population measures and the number of households of all intersections that belong to a circular buffer. Lastly, using the circular-buffer demographic information, we calculate the demographic attributes of the buffers for our analysis.

Table 2 shows the frequency distribution of facility-year observations for the occurrence of excess emissions. During the study period, more than 60% of the facility-year observations have no excess emissions. The excess emissions data, moreover, are unevenly distributed, and specifically skewed to the right. The mean is around 68 pounds, while the maximum is almost 300,000 pounds (standard deviation is 2,300). (See **Table B** in **S1 File** for the descriptive statistics of the independent variables used in the facility level analysis.)

**Table 2:** Facility excess emissions status.

Facility Excess Emissions Status	Frequency	Percent
1 (Excess Emissions > 0)	8,441	39.42%
0 (Excess Emissions = 0)	12,974	60.58%
Total	21,415	100.00%

Note: Values in Frequency column represent the number of facility-year observations.

#### Advantages and limitations of statistical methods

The census tract and facility-level analyses provide alternative, but complementary, approaches to examining the relationship between excess emissions and demographics. Given the structure of the data, each approach has advantages and limitations.

An important advantage of the multinomial model at the census tract level is that it avoids concerns about selection bias that may arise from only considering cases of positive excess emissions. That is, if we were to study the association between demographic attributes and the level of excess emissions only in places with these emissions occur, the analysis would fail to account for possible unobserved correlation between the *location* of polluting facilities and demographics (e.g., if firms’ siting decisions are related to the demographic composition of host areas). The census tract analysis includes all census tracts in Texas, which implicitly takes into account any such correlation (measured by the existence of routine emissions). The census tract approach also mitigates the concern about the heavily skewed distribution of excess emissions by categorizing census tracts into three types, rather than modeling emissions directly.

The census tract approach does have some limitations, however. First, this approach ignores the variation in the level of excess emissions, which may contain important information about the relationship between excess emissions and demographics. Second, the census tract analysis does not enable us to control for any facility characteristics, and may mask important factors in explaining excess emissions. Last, this approach assumes that emissions from a facility only affect the census tract that hosts the facility, which ignores the fact that emissions released from a facility may affect other nearby census tracts. This is especially likely if the facility is near a tract border or the area has high population density (and thus the geographic size of census tracts are relatively small), and in cases when the pollutant travels long distances affecting communities in downwind census tracts (or even different downwind counties or states).

The facility-level analysis helps addresses many of these concerns. By modeling the relationship between demographics and, first, whether a facility has excess emissions (the logit model) and then the levels of these emissions (the OLS model), we can better understand the patterns of any correlations. Moreover, using two models allows us to address the skewness of the underlying data and specifically offers a way to model the mass of zeros while still preserving the variation in the level of excess emissions. The facility-level analysis also allows us to control for some facility characteristics, and specifically the size and industrial type of the facility, which may be an important omitted attribute in the census tract analysis. Finally, in facility level analysis, we can more carefully delineate the neighborhoods potentially most affected by excess emissions, thereby accounting for cases where facilities are located at the border of multiple tracts or in areas of high population density.

There are three important limitations of modeling the relationship between demographics and excess emissions at the facility level. First, the analysis does not fully resolve the issue of emissions that spill over into other areas. This is especially true for instances of long-range transport of pollutants. Second, the location decision of facilities pre-dates the occurrence of excess emissions, and our analysis thus incorporates any residential sorting that occurred in the period after the siting of the facility. As a result, we cannot draw any causal interpretations or make generalize inferences to the entire state of Texas from the analysis, since there are large parts of Texas without any facilities that may have very different demographics. Last, in the OLS model of excess emissions levels, we condition on the occurrence of excess emissions. Thus, our conclusions are only relevant to this subcategory of facilities, since there might be a demographic disparities in where excess emissions occur in the first place.

In sum, the census tract and facility level analyses each have limitations. But, to the extent that the findings from each analysis complement each other, they jointly provide a stronger footing for characterizing the relationships between demographics and excess emissions.

## Results

We discuss results, first for the census tract level analysis and subsequently for the facility level analysis.

### Census tract level findings

The multinomial regression coefficients are presented in **Table 3**. The results show that statistically significant correlations exist between demographics and census tract types. The log odds ratio of being a type 2 census tract against being a type 1 census tract (i.e., a tract with routine, but no excess emissions compared to tract with no emissions at all) increases as the percentage of Black population increases and the percentage of Hispanic population decreases. This log odds ratio decreases as population density increases, percentage of college graduate increases, median household income decreases, median household income decreases, median housing value increases, and percentage of owner-occupied housing unit increases. This pattern of relationship generally applies to the comparison between type 3 tract and type 1 tract (i.e., a tract with routine and excess emissions compared to tract with no emissions at all) as well, except that there is no significant association between the percentage of Hispanic population and the log odds of type 3 against type 1.

**Table 3:** Census tract level analysis multinomial model coefficients.

Multinomial Model: Logit Coefficients		
	Type 2 | Type 1	Type 3 | Type 1
%Black (0–100)	0.0061**	0.0035**
	(0.0010)	(0.0015)
%Hispanic (0–100)	-0.0021**	0.0005
	(0.0008)	(0.0008)
Pop Density (Pop/Acre)	-0.3230**	-0.7480**
	(0.0078)	(0.0185)
%College Graduate (0–100)	-0.0278**	-0.0437**
	(0.0022)	(0.0032)
Median Household Income ($1,000)	0.0120**	0.0337**
	(0.0018)	(0.0024)
Median Housing Value ($1,000)	-0.00066	-0.0071**
	(0.0005)	(0.0008)
% Owner-occupied Housing Unit (0–100)	-0.0230**	-0.0218**
	(0.0012)	(0.0016)
Constant	0.5483**	-0.0296
	(0.0968)	(0.1358)
N	56,536	56,536

Note: (1) Standard errors in parentheses. Statistical significance levels

* *p* < 0.10

** *p* < 0.05.

(2) Type 1 (Routine Emissions = 0, Excess Emissions = 0); Type 2 (Routine Emissions > 0, Excess Emissions = 0); Type 3 (Routine Emissions > 0, Excess Emissions > 0)

**Fig 1:**
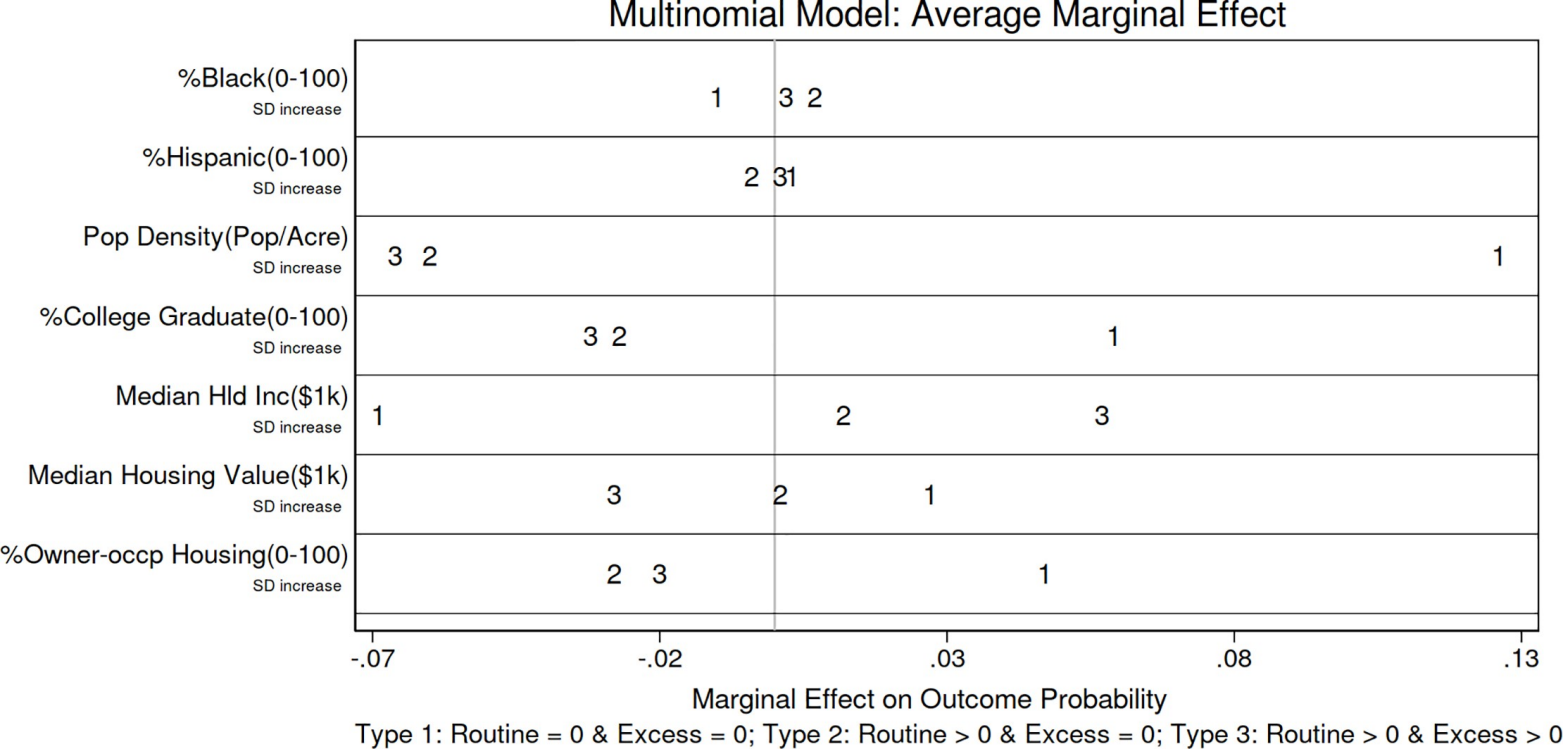
Multinomial logit average marginal effects.

To provide a more intuitive interpretation of the multinomial logit results, we calculate the average marginal effect (AME) for each variable. The AME takes all pairwise regression results into consideration, calculates the marginal effect for each observation and takes the average. It shows the relationships between the independent variables and the probabilities of being different types of census tract. **Fig 1** displays the average marginal effects to facilitate comparison (see **Table C** in **S1 File** for the actual AME values).

**Fig 1** shows that census tracts with a higher percentage of Black population have higher probabilities of having routine emissions (being type 2 or type 3) and excess emissions (being type 3). A one standard deviation (17 percentage point) increase in the share of Black population is associated with a 0.009 increase in the probability of having routine emissions and a 0.002 increase in the probability of having excess emissions. Given the low probability for a census tract to have routine emissions (being type 2 or type 3) and the low probability to have excess emissions (being type 3) in the first place, the increases (that associate with a one standard deviation increase in the percentage of Black population) represent a 6% increase of the probability of having routine emissions and a 3% increase of the probability of having excess emissions respectively.

Population density is a strong predictor of pollution as well. One standard deviation (5 persons/acre) increase in population density correlates with a 0.126 decrease (an 81% decrease) in the probability of having routine emissions and a 0.066 decrease (a 96% decrease) in the probability of having excess emissions. The share of college graduate is also strongly correlated with having routine and excess emissions. A standard deviation (18 percentage points) increase in the share of college graduate is associated with a decrease of the probability of having routine emissions by 0.059 (a 38% decrease) and the probability of having excess emissions by 0.032 (a 46% decrease). With respect to median household income, a one standard deviation ($25,000) increase is associated with a 0.069 increase (a 45% increase) in the probability of having routine emissions and a 0.057 increase (an 83% increase) in the probability of having excess emissions. The relationship between median housing value and the probability of being type 2 (routine emissions > 0 but excess emissions = 0) census tract is not significant, while a one standard deviation ($ 86,000) increase of median housing value is correlated with a 0.028 decrease (a 41% decrease) in the probability of being type 3 (routine emissions >0 and excess emissions>0) census tract. Census tracts with higher percentage of owner-occupied housing unit have lower probabilities of having routine emissions (being type 2 or type 3) and excess emissions (being type 3). A one standard deviation (21 percentage point) increase in the percentage of owner-occupied housing is associated with a 0.048 decrease (a 31% decrease) in the probability of having routine emissions and a 0.020 decrease (a 29% decrease) in the probability of having excess emissions.

As a check on the robustness of these findings, we also estimated 4 and 5 category models, which categorize census tracts into more types. The 4 category model divides the type 3 (Routine emissions >0, Excess emissions >0) census tracts into two types based on whether regulatory penalties were assessed on excess emissions. The 5 category model divides the type 3 (Routine Emissions > 0, Excess Emissions > 0) census tracts into three types based on subtypes of excess emissions: 1) Routine emissions >0, SMSS > 0, EE = 0; 2) Routine emissions >0, SMSS = 0, EE >0; 3) Routine emissions >0, SMSS >0, EE>0. The rationale for considering 4 and 5 category models is that excess emissions can differ in important ways that could relate to their association with demographic attributes. The results from the 4 and 5 category models are generally consistent with what we found for the 3 category model; the demographic attributes have similar correlations with subtypes of excess emissions, as they do with excess emissions as a whole. We report these results in **Table D** in **S1 File, Figure A** in **S1 File, Table E** in **S1 File**, and **Figure B** in **S1 File**.

### Facility level findings

Our second approach focuses on the facility as the unit of observation and considers a set of demographic characteristics in a 1-mile buffer around each facility. The analysis consists first of a logit model then a second conditional OLS model. **Table 4** presents the average marginal effects from three model specifications, which differ only in the included fixed effects. The first specification includes industry and year fixed effects, the second specification adds county fixed effects, and the third specification includes county-year fixed effects.

**Table 4:** Facility level analysis (1-mile buffer).

	Facility Level: Logit Model and OLS Model AME					
	(1)		(2)		(3)	
	Logit	OLS	Logit	OLS	Logit	OLS
% Black (0–100)	-0.0005	0.0107**	0.0000	0.0096**	-0.0003	0.0101**
	(0.0003)	(0.0032)	(0.0004)	(0.0037)	(0.0004)	(0.0040)
% Hispanics (0–100)	0.0008**	0.0031*	0.0010**	-0.0007	0.0009**	-0.0018
	(0.0002)	(0.0017)	(0.0003)	(0.0034)	(0.0004)	(0.0036)
Ln (Pop Den)	-0.0171**	-0.114**	-0.0163**	-0.0633*	-0.0174**	-0.0680*
	(0.0021)	(0.0200)	(0.0036)	(0.0345)	(0.0036)	(0.0362)
% College Grad (0–100)	-0.0006	-0.0142**	0.0004	-0.0158**	0.0009	-0.0178**
	(0.0005)	(0.0051)	(0.0006)	(0.0067)	(0.0006)	(0.0072)
Ln (Md Hld Inc)	0.0992**	1.3160**	0.0970**	1.1410**	0.0493	1.2320**
	(0.0204)	(0.2030)	(0.0295)	(0.2960)	(0.0322)	(0.3430)
Ln (Md Hous Val)	-0.0227*	-0.7520**	-0.0414**	-0.5570**	-0.0306	-0.5810**
	(0.0132)	(0.1310)	(0.0199)	(0.2040)	(0.0219)	(0.2400)
% Owner-occ (0–100)	-0.0014**	0.0023	-0.0015**	-0.0132**	-0.0012**	-0.0153**
	(0.0003)	(0.0031)	(0.0004)	(0.0039)	(0.0004)	(0.0043)
Ln (Routine Emissions)	0.0660**	0.5260**	0.0602**	0.5090**	0.0650**	0.5270**
	(0.0019)	(0.0173)	(0.0020)	(0.0173)	(0.0021)	(0.0185)
Industry Fixed Effects	X	X	X	X	X	X
Year Fixed Effects	X	X	X	X		
County Fixed Effects			X	X		
County by Year Fixed Effects					X	X
N	20,316	8,436	20,170	8,436	18,636	8,436

Note: Standard errors in parentheses. Statistical significance levels

* *p* < 0.10

** *p* < 0.05.

The results from the facility level analysis show similar relationships between demographics and excess emissions as those of the census tract analysis. Across our three model specifications, the logit results indicate that facility-neighborhoods with or without excess emissions have no discernable difference in the share of Black population. However, conditional on having some amount of excess emissions, facility-neighborhoods with a higher share of Black population, tend to have higher excess emission amounts. Facility-neighborhoods with excess emissions tend to have higher shares of Hispanic population, though the level of emissions is associated with the percentage of Hispanics in only one model. Facility-neighborhoods with higher population density are less likely to have excess emissions and, once excess emissions occurred, tend to have lower amounts of excess emissions. Facility-neighborhoods with or without excess emissions show no significant difference in the percentage of college graduate while conditional on having excess emissions, facility-neighborhoods with a higher share of college graduate tend to have smaller amount of excess emissions.

Consistent with the results from the tract level analysis, the facility level analysis shows that facility-neighborhoods with higher median household incomes are more likely to have excess emissions and conditional on having excess emissions, also are more likely to have higher levels of excess emissions. Facility neighborhoods with lower median housing values and with lower percentages of owner-occupied housing are more likely to have excess emissions and, conditional on occurrence of excess emissions, tend to have more excess emissions. Facility neighborhoods with more routine emissions tend to have excess emissions and conditional on that, have higher excess emission amounts.

As check on the robustness of these findings, we also estimated the model using a 3-mile buffer to compute the demographic features of facilities. The results are generally consistent with these of the 1-mile buffer analysis. **Table F** in **S1 File** presents these results.

## Discussion

In this paper, we analyze the correlations between excess emissions and demographic characteristics at the census tract level with a multinomial modeling approach and at the facility level with a logit and a conditional OLS modeling approach. Results from both approaches show that the geographical distribution of excess emissions is not random regarding neighborhoods’ racial, ethnic, and socioeconomic attributes. Specifically, we have found that excess emissions have a positive association with the percentage of Black population. The relationship between excess emissions and the percentage of Hispanic population is less consistent, but provide some suggestive evidence of positive correlation as well. Excess emissions are found to be strongly negatively correlated with population density and the percentage of college graduate. In addition, we have found negative correlations between excess emissions and median housing value and the percentage of owner-occupied housing. However, we also find a strong positive correlation between median household income and excess emissions.

These results are generally consistent with existing evidence of disparities in allocation of environmental burdens, although our findings pertaining to income run counter to typical expectations rooted in the environmental justice literature [43–46]. These counterintuitive results may arise from the fact that, while facilities create pollution, they may also positively contribute to the economic activities in their neighborhoods. This explanation illustrates the complexities in the relationship between economic welfare and pollution, and there are numerous studies that have found that the relationship between pollution and income is positive [47, 48] or curvilinear [49–53]. It is also possible that these results regarding income are due to idiosyncrasies in facility siting patterns and the distribution of excess emissions in Texas. Additional studies that focus on excess emissions at the national level or in other regions are needed to further shed light on this issue.

While we believe this research suggests important disparities in the distribution of excess emissions in Texas, the results should be interpreted with some caution given several methodological caveats. First, our analyses are correlational, and cannot (nor do we claim otherwise) establish a causal explanation for the observed distribution of excess emissions. The pattern of the distribution of excess emissions may result from households’ residential choices, the siting of polluting facilities in minority neighborhoods, or unobserved facility-level characteristics such as local weather conditions, infrastructure quality, regulatory pressure, or poor operation and management practices. Previous research on the causes of disparate distribution of pollution suggests that they may result from a combination of these factors. For instance, the literature has documented inequalities in the facility siting decisions [31, 54], demographic changes after siting of facilities [31], and weaker enforcement of environmental regulations in minority communities [55–58]. Future work is needed to disentangle the causes for the disparities in the distribution of excess emissions.

The second caveat is that our research does not capture population’s exposure to excess emissions and the ensuing health impacts. In this study, we use population’s proximity to the sources of excess emissions as a proxy for exposure. Our analyses do not take into consideration factors such as the fate and toxicity of different pollutants and the transport of excess emissions in the environment. As a result, we cannot infer anything about the distribution of exposure to and risk associated with excess emissions. While challenging, understanding the distribution of exposure and risk is critical to more accurately assess the impact of excess emissions. Several scholars [45, 53, 56] have attempted to better characterize the nature of exposure and risk of pollution using tools such as EPA’s Risk-Screening Environmental Indicators modesl. Similar efforts to more accurately capture different population’s exposure to excess emissions and the resulting risk and health impacts are needed.

The third caveat is that our statistical models analyzed different types of study samples, which limits the reach of our inferences. Specifically, the census tract level analysis included all neighborhoods in Texas, regardless of the presence of facilities or excess emissions. Hence, results from this analysis are applicable to the whole state of Texas. However, in the facility level analysis, we narrow the universe of the study population. The logit model includes only neighborhoods with facilities, so the results can only be generalized to areas with facilities. The OLS model further narrows down the population to neighborhoods with facilities that have excess emissions. Results from this analysis can only be applied to such neighborhoods. On this note, results from our multiple models do tend to complement each other, which offers more robustness to our investigation of the disparities of the distribution of excess emissions.

Despite these caveats, this study contributes new empirical evidence to the environmental justice literature. As the first study to document the racial, ethnic, and income disparities of excess emissions, it broadens the literature to a new category of air pollution. The study also adds to our understanding of the implication of excess emissions. In particular, our findings highlight which segments of the population may be most affected by the historical under-regulation of this type of industrial pollution. To the extent that the cost of excess emissions–in terms of adverse health impacts, or quality of life more generally–is disproportionately placed on neighborhoods with large proportions of people of color whose residents are already often subject to disproportionate environmental burdens, the findings of this research add to the urgency of legislative and administrative efforts to regulate excess emissions.

## Supporting information

S1 File (PDF)
