# Supplementary material for: Racial, ethnic, and income disparities in air pollution: A study of excess emissions in Texas
Source: PLoS One. 2019 Aug 2;14(8):e0220696. doi: 10.1371/journal.pone.0220696 (PMC6677308; doi:10.1371/journal.pone.0220696)
Supplement: S1 File — (PDF) [file pone.0220696.s001.pdf]

Table A: Descriptive Statistics of Census Tract-Year Demographic Variables

	Census Tract Type					
	Type 1 (N = 47,746)		Type 2 (N = 4,880)		Type3 (N =3910)	
Statistics	Mean	S.D.	Mean	S.D.	Mean	S.D.
% Black (0-100)	11.60	17.02	13.74	18.11	9.61	13.90
% Hispanics (0-100)	34.68	28.41	29.88	25.22	29.34	24.84
Population Density (per acre)	4.97	4.65	1.89	2.69	0.67	1.43
% College Graduate (0-100)	25.37	18.88	18.67	14.03	15.80	10.80
Median Household Income ($1,000)	50.10	26.34	43.71	18.27	44.08	15.44
Median Housing Value ($,1000)	119.84	90.34	95.13	55.67	86.81	46.96
% Owner-occupied Housing Unit (0-100)	64.53	21.76	66.33	20.80	73.43	15.42

Table B: Descriptive Statistics of Emissions and Facility-Year Demographic Variables

	Facility Type			
	Facility with Excess Emissions = 0 (N = 12,974)		Facility with Excess Emissions > 0 (N = 8,441)	
Statistics	Mean	S.D.	Mean	S.D.
% Black (0-100)	11.67	14.40	8.60	12.61
% Hispanics (0-100)	29.86	23.23	31.88	23.90
Population Density (per acre)	1.30	2.06	0.61	1.25
% College Graduate (0-100)	17.00	11.38	14.89	9.21
Median Household Income ($1,000)	44.50	15.78	44.84	14.04
Median Housing Value ($,1000)	92.44	48.54	85.65	40.55
% Owner-occupied Housing (0-100)	68.56	17.52	72.40	13.60
Routine Emissions (pounds)	2974.78	145057.60	29143.09	524962.20
Excess Emissions (pounds)			173.44	3669.09

Note: Excess emissions consist of EE emissions and SMSS emissions. Facilities started to report them separately to EI from 2004. Thus,breakdown of excess emissions into the two categories for the entire 2000 – 2010 period is not possible. For the period 2004 – 2010, facility-year observations with positive excess emissions have average EE emissions of 102 pounds (s.d. = 1,800) and average SMSS emissions of 81 pounds (s.d. = 3052).

Table C: Multinomial Logit Average Marginal Effects (3-type Categorization of Census Tract-Year Observations)

Average Marginal Effects				
		Pr (Type 1)	Pr (Type 2)	Pr (Type 3)
% Black (0-100)				
	+ SD	-0.010	0.007	0.002
	p-value	0.000	0.000	0.113
% Hispanic (0-100)				
	+ SD	0.003	-0.004	0.001
	p-value	0.095	0.003	0.294
Population Density (per acre)				
	+ SD	0.126	-0.060	-0.066
	p-value	0.000	0.000	0.000
% College Graduate (0-100)				
	+ SD	0.059	-0.027	-0.032
	p-value	0.000	0.000	0.000
Median Household Income ($1,000)				
	+ SD	-0.069	0.012	0.057
	p-value	0.000	0.002	0.000
Median Housing Value ($1,000)				
	+ SD	0.027	0.001	-0.028
	p-value	0.000	0.810	0.000
% Owner-occupied Housing Unit (0-100)				
	+ SD	0.047	-0.028	-0.020
	p-value	0.000	0.000	0.000

Table D: Multinomial Logit Average Marginal Effects (4-type Categorization of Census Tract-Year Observations)

Average Marginal Effects					
		Pr (Type 1)	Pr (Type 2)	Pr (Type 3)	Pr (Type 4)
% Black (0-100)					
	+ SD	-0.010	0.007	0.002	0.001
	p-value	0.000	0.000	0.218	0.291
% Hispanic (0-100)					
	+ SD	0.003	-0.004	-0.001	0.002
	p-value	0.109	0.003	0.578	.
Population Density (per acre)					
	+ SD	0.126	-0.060	-0.055	-0.011
	p-value	0.000	0.000	0.000	0.000
% College Graduate (0-100)					
	+ SD	0.057	-0.027	-0.021	-0.009
	p-value	0.000	0.000	0.000	0.000
Median Household Income ($1,000)					
	+ SD	-0.080	0.009	0.022	0.049
	p-value	0.000	0.017	0.000	0.000
Median Housing Value ($1,000)					
	+ SD	0.027	0.001	-0.022	-0.006
	p-value	0.000	0.836	0.000	0.000
% Owner-occupied Housing Unit (0-100)					
	+ SD	0.047	-0.028	-0.014	-0.005
	p-value	0.000	0.000	0.000	0.000

Figure A: Multinomial Logit Average Marginal Effects (4-type Categorization of Census Tract-Year Observations)

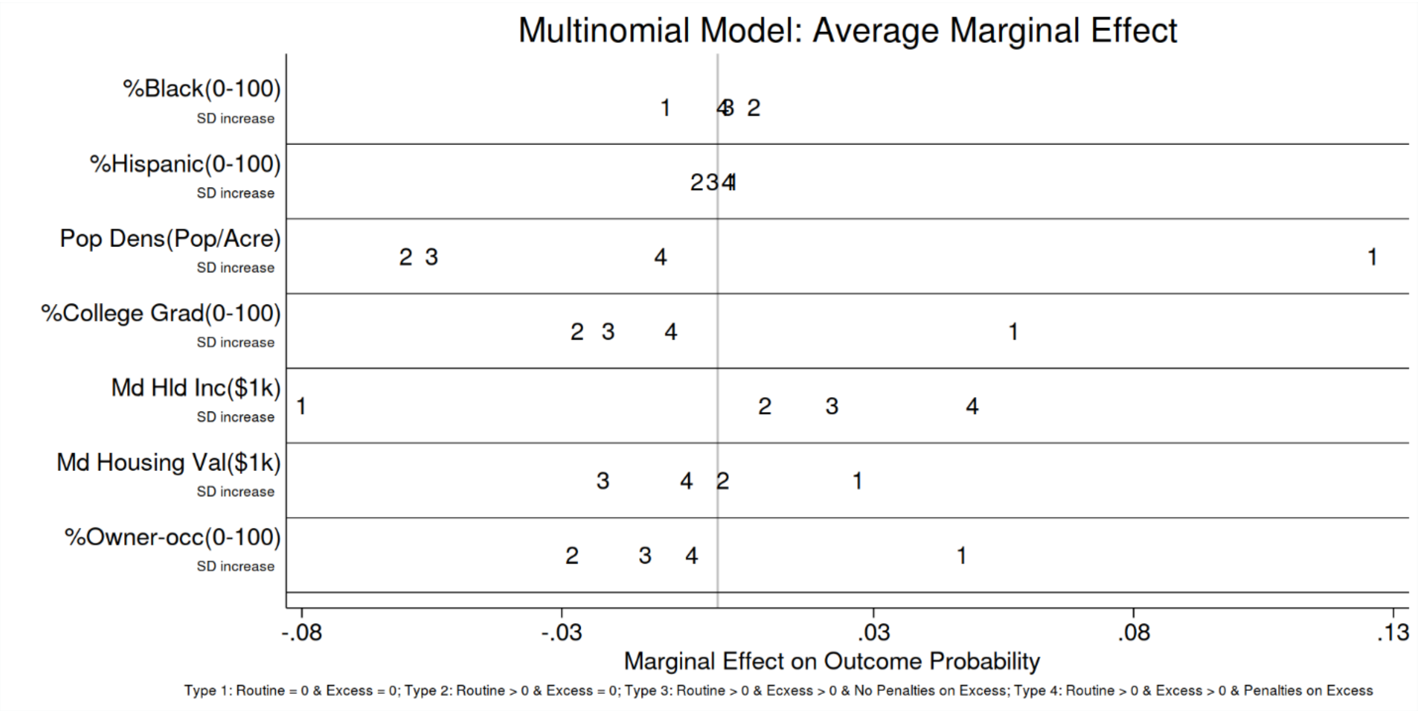

Table E: Multinomial Logit Average Marginal Effects (5-type Categorization of Census Tract-Year Observations)

Average Marginal Effects					
	Pr (Type 1)	Pr (Type 2)	Pr (Type 3)	Pr (Type 4)	Pr (Type 5)
% Black (0-100)					
+ SD	-0.011	0.008	0.001	0.003	-0.001
p-value	0.000	0.000	0.138	0.001	0.424
% Hispanic (0-100)					
+ SD	0.000	-0.004	-0.002	-0.001	0.007
p-value	0.985	0.026	0.016	0.386	0.000
Population Density (per acre)					
+ SD	0.127	-0.057	-0.017	-0.014	-0.038
p-value	0.000	0.000	0.000	0.000	0.000
% College Graduate (0-100)					
+ SD	0.059	-0.023	-0.010	-0.006	-0.021
p-value	0.000	0.000	0.000	0.000	0.000
Median Household Income ($1,000)					
+ SD	-0.082	0.010	0.005	0.013	0.053
p-value	0.000	0.021	0.083	0.000	0.000
Median Housing Value ($1,000)					
+ SD	0.026	0.000	-0.001	-0.006	-0.019
p-value	0.000	0.965	0.651	0.000	0.000
% Owner-occupied Housing Unit (0-100)					
+ SD	0.048	-0.026	-0.002	-0.007	-0.013
p-value	0.000	0.000	0.166	0.000	0.000

Figure B: Multinomial Logit Average Marginal Effects (5-type Categorization of Census Tract-Year Observations)

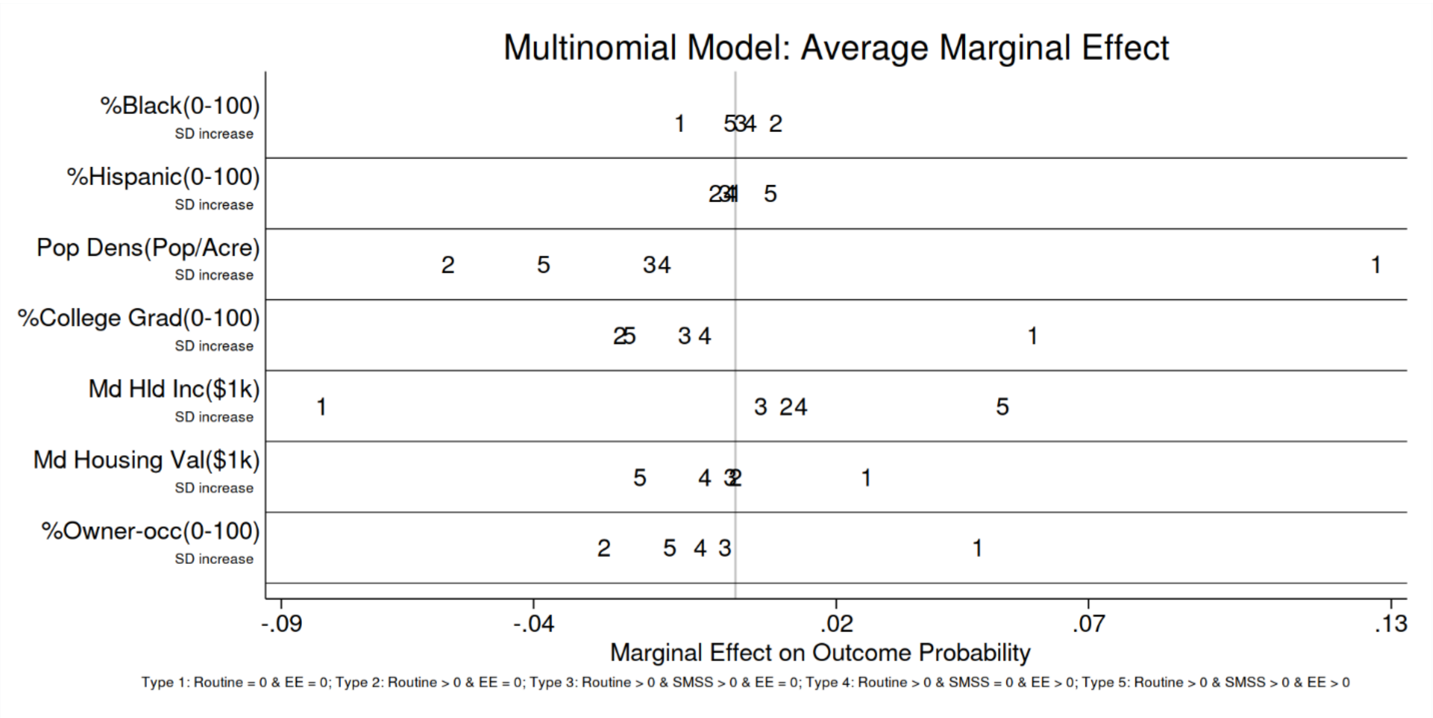

Table F: Facility Level Analysis (3-mile Buffer)

	Facility Level: Logit Model and OLS Model AME					
	(1)		(2)		(3)	
	Logit	OLS	Logit	OLS	Logit	OLS
% Black (0-100)	-0.0004	0.0190**	0.0001	0.0151**	-0.0003	0.0149**
	(0.0003)	(0.0031)	(0.0004)	(0.0041)	(0.0004)	(0.0044)
% Hispanics (0-100)	0.0007**	0.0058**	0.0006	-0.0017	0.0003	-0.0045
	(0.0002)	(0.0017)	(0.0004)	(0.0040)	(0.0004)	(0.0043)
Ln (Population Density)	-0.0158**	-0.1140**	-0.0117**	-0.1010**	-0.0112**	-0.0932**
	(0.0022)	(0.0216)	(0.0040)	(0.0410)	(0.0041)	(0.0435)
% College Grad (0-100)	-0.0011**	-0.0107*	-0.0003	-0.0133*	0.0002	-0.0187**
	(0.0005)	(0.0055)	(0.0008)	(0.0075)	(0.0008)	(0.0081)
Ln (Md Hld Inc)	0.1080**	1.5240**	0.0934**	1.4790**	0.0137	1.5380**
	(0.0225)	(0.2160)	(0.0361)	(0.3470)	(0.0416)	(0.4140)
Ln (Md Housing Val)	-0.0234	-0.8000**	-0.0224	-0.4470*	0.0005	-0.427
	(0.0151)	(0.1520)	(0.0237)	(0.2420)	(0.0266)	(0.2830)
% Owner-occ Hou (0-100)	-0.0021**	0.0080**	-0.0020**	-0.0143**	-0.0015**	-0.0166**
	(0.0004)	(0.0040)	(0.0005)	(0.0055)	(0.0006)	(0.0061)
Ln (Routine Emissions)	0.0669**	0.5200**	0.0607**	0.5130**	0.0656**	0.5330**
	(0.0019)	(0.0174)	(0.0020)	(0.0174)	(0.0021)	(0.0185)
Industry Fixed Effects	X	X	X	X	X	X
Year Fixed Effects	X	X	X	X		
County Fixed Effects			X	X		
County by Year Fixed Effects					X	X
N	20,349	8,439	20,203	8,439	18,669	8,439

Note: Standard errors in parentheses. Statistical significance levels: * $p < 0.10$, ** $p < 0.05$
